# Self-harm amongst people of Chinese origin versus White people living in England: a cohort study

**DOI:** 10.1186/s12888-015-0467-0

**Published:** 2015-04-14

**Authors:** Shu-Sen Chang, Sarah Steeg, Navneet Kapur, Roger T Webb, Paul SF Yip, Jayne Cooper

**Affiliations:** Institute of Health Policy and Management, and Department of Public Health, College of Public Health, National Taiwan University, No 17, Xuzhou Road, Taipei, 10055 Taiwan; The Hong Kong Jockey Club Centre for Suicide Research and Prevention, The University of Hong Kong, 5 Sassoon Road, Pokfulam, Hong Kong, SAR China; Centre for Mental Health and Safety, University of Manchester, Jean McFarlane Building, Oxford road, Manchester, M13 9PL UK

**Keywords:** Self-Harm, Chinese, Ethnicity, Epidemiology

## Abstract

**Background:**

There has been little previous research on self-harm among people of Chinese origin living in the UK, although this population has grown substantially in recent years and China is now the largest source of international students at UK universities.

**Methods:**

We conducted a prospective cohort study using self-harm presentation data (1997–2011) collected from three hospitals in the City of Manchester, which has the largest Chinese population across all UK Local Authorities. Rate ratios between the Chinese and White groups were calculated using Poisson regression models. Chi-square tests (or Fisher’s exact tests), logistic regression, and log-binomial regression were used to examine differences in characteristics and clinical management between groups.

**Results:**

Ethnicity was known in the study cohort for 23,297 (87%) amongst 26,894 individuals aged 15 years and above. A total number of 97/23,297 (0.4%) people of Chinese ethnic origin presented with self-harm over the study period and 20,419 (88%) were White people. Incidence of self-harm in the Chinese group (aged 16–64 years) was less than one fifth of that found in White people (0.6 versus 3.2 per 1000 person-years; rate ratio 0.18, 95% confidence interval 0.13-0.24), and was particularly low amongst men of Chinese origin. Individuals of Chinese origin who presented with self-harm were younger, more likely to be female and students, and more likely to self-injure and describe relationship problems as a precipitant than White people. They were less likely to have clinical risk factors such as drug/alcohol misuse and receiving psychiatric treatment, and were rated to have lower risk of self-harm repetition by treating clinicians.

**Conclusion:**

Future research needs to investigate whether the low incidence of self-harm presenting to hospitals amongst people of Chinese origin truly reflects a lower frequency of self-harm, or alternatively is due to markedly different post-episode help-seeking behaviours or student overrepresentation in this ethnic group. Relevant healthcare professionals need to be aware of the risk characteristics of people of Chinese origin who self-harm.

**Electronic supplementary material:**

The online version of this article (doi:10.1186/s12888-015-0467-0) contains supplementary material, which is available to authorized users.

## Background

According to the 2011 census, the size of the Chinese population in England and Wales was approximately 393,000, a 127% increase compared to data from 1991 [1]. People of Chinese origin accounted for 5% of all ethnic minority populations and 0.7% of the total population of England and Wales in 2011 [1]. China is now the largest source of international students at UK universities; Chinese students studying at UK higher education institutions totalled around 79,000 in 2011–2012, accounting for 26% of non-EU students [2]. However, research evidence regarding psychological wellbeing and suicidal behaviour in this group is scarce, although it was reported that China had a relatively high suicide rate [3] and during the stages of migration there could be factors that predispose individuals to mental illness and suicidal behaviour [4].

Previous studies of suicidal behaviour amongst minority ethnic groups in the UK have focussed on people of South Asian origin or black people [5-7]. A recent study in England and Wales showed that suicide rates were higher in older women, but lower in younger men who were first generation migrants from China compared with locally born people [6]. There has been no specific investigation to date of the incidence and characteristics of self-harm amongst people of Chinese origin in the UK.

The aims of this study were to compare people of Chinese origin and White people with regard to overall and sex- and age-specific incidence of self-harm; socio-demographic and clinical characteristics; clinical management following self-harm; and risk of self-harm repetition. The study setting was the City of Manchester, which has the largest absolute number of Chinese origin residents across all UK Local Authority areas.

## Methods

### Data

We conducted a prospective cohort study using data from the Manchester Self-Harm (MaSH) project, of people presenting to three study hospitals providing emergency care in the Northwest of England (UK) during 1st September 1997 to 31st December 2011. The MaSH project was established in 1997 with an aim to monitor hospital presentations of self-harm and its methodology has been described in detail elsewhere [8,9]. Briefly, data were extracted from a standard patient assessment form that was completed by treating clinicians (emergency department or psychiatric staff) to record information on demographic and clinical characteristics, details of the self-harm episode, risk assessment, and follow-up arrangements. From 1^st^ September 2002, basic information was also collected by the research team from the medical records for patients who were not assessed by clinicians (for example, because they did not wait or refused assessment). Self-harm was defined as intentional self-poisoning or self-injury, irrespective of motivation and degree of suicidal intent [10]. Ethnicity for each patient was ascribed by the treating clinician or hospital staff at time of admission according to standard UK national 2001 Census categories or later assigned based on information from medical records. For analytical purposes we extracted data for two groups: people of Chinese origin (including second and third generation individuals, as well as people born in China) and White people (White British, Irish, or White Other).

Regarding methods used in self-harm, the most lethal method was coded as the primary method when multiple methods were used (only 3-4% of all self-harm episodes [11]); for example, self-poisoning would be coded over cutting. For the characteristics of self-harm, including precipitant factors, clinical characteristics, symptoms of depression and circumstances of episode, data were obtained from the specialist psychiatric assessment. The assessing clinician would have categorised each factor, characteristic, symptom, or circumstance as present or not present and recorded this on the form. If there was no psychiatric assessment, the information was then extracted from an assessment carried out by an Emergency Department clinician as possible.

The three study hospitals have near to complete coverage of the City of Manchester population, i.e. if a resident of the City harms herself/himself they are likely to attend the emergency department of these three hospitals if they do attend a hospital. A previous local audit of patient ‘cross-flows’ showed that only a small minority of residents visited hospitals outside of the three study hospitals, and we estimated that we captured over 90% of self-harm presentations [12]. In 2011 the city had a population of 503,127, of whom 2.7% (n = 13,539) were of Chinese origin; this is the largest Chinese population across all UK Local Authorities (local government body), and the percentage of Chinese population in the city was amongst the highest in the UK [13]. Population data for the City of Manchester, stratified by age, sex, and ethnicity, were obtained from 2001 and 2011 UK Censuses [13,14]. In Manchester the Chinese population aged 16–64 grew 2.6 times during 2001–2011, from around 4,400 to 11,400.

### Ethical considerations

The MaSH project has been ratified as part of a clinical audit system by local research ethics committees, and the NHS. Thus, formal ethics committee approval was not required. The MaSH project is fully compliant with the UK Data Protection Act 1998, and has support under Section 251 of the NHS Act 2006 regarding the use of patient-identifiable data.

### Statistical analysis

We conducted two sets of analyses based on each individual’s first presentation for self-harm during the study period (their ‘index episode’). First, we calculated incidence of self-harm per 1000 person-years for individuals aged 16–64. This age range was chosen to be comparable to previous analyses [15,16], and for pragmatic reasons as there were only two elderly men of Chinese origin outside this age range. This analysis was based on data for patients with a City of Manchester postcode during September 2002 to December 2011, when we had complete data for all self-harm presentations including both assessed and non-assessed individuals. Approximate person-years at risk were estimated using sex-, age- and ethnic group-specific population data from the 2001 and 2011 Censuses, and linear projections for the years between Censuses. Rate ratios between people of Chinese origin and White people (and their 95% confidence intervals) were calculated using Poisson regression models. The outcome variable was the number of people who self-harmed, with census-derived person-years estimates as the offset, and the exposure variable was ethnicity (Chinese versus White people) in Poisson regression models. There was no statistical evidence for overdispersion in these models.

Second, individuals aged 15 or above who presented to study hospitals with self-harm between 1^st^ September 1997 and 31^st^ December 2011 were investigated, regardless of area of residence. In these analyses, chi-square tests, or Fisher’s exact tests where appropriate, were used to examine differences in socio-demographic characteristics, method of self-harm, precipitating factors, and clinical characteristics between the two ethnic groups. In a sensitivity analysis we estimated the odds ratios of various characteristics for the Chinese group (White people as the control/comparison group), before and after adjusting for sex and age, using logistic regression models. Log-binomial regression was used to estimate risk ratios, unadjusted and sex-and-age-adjusted, between the two groups for differential percentages in clinical management and self-harm repetition outcomes. In our self-harm repetition analyses we excluded individuals who first self-harmed in 2011, as we could not ensure a full 12-month follow-up period. All analyses were conducted using IBM SPSS Statistics for Windows, Version 20.0 (IBM Corp., Armonk, NY, 2011) and Stata version 13 (StataCorp, College Station, TX, 2013).

## Results

**Figure 1:**
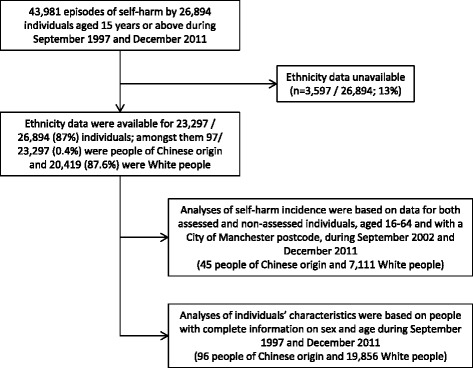
Flow chart of study samples.

During the study period we observed 43,981 episodes of self-harm by 26,894 individuals aged 15 years or above. Ethnicity data were available for 23,297/26,894 (87%) individuals, amongst whom 97/23,297 (0.4%) were of Chinese origin and 20,419 (87.6%) were White people. Figure 1 shows a flow chart summarising the study samples.

### Incidence of self-harm

Estimation of self-harm incidence was based on 45 people of Chinese origin (10 males, 35 females) and 7,111 White people (3,130 males, 3,981 females) (Figure 1 and Table 1). Self-harm incidence was much lower in people of Chinese origin (0.6 per 1000 person-years) than in White people (3.2 per 1000 person-years), with a rate ratio of 0.18 (95% confidence interval [CI] 0.13-0.24); the pattern was similar amongst individuals aged 16–34 and 35–64 whilst the rate ratios appeared to be lower in males (0.09, 95% CI 0.05-0.17) than in females (0.24, 95% CI 0.17-0.34), with a statistically significant sex difference observed (interaction: P = 0.006). Men of Chinese origin had much lower incidence of self-harm than their female counterparts (0.3 versus 0.9 per 1000 person-years); the female-to-male ratio amongst people of Chinese origin was 3.5 (95% CI 1.7-7.0), compared to 1.3 (95% CI 1.2-1.4) in White people.

**Table 1:** **Age**, **sex and ethnic group-specific self-harm incidence (per 1000 person-years) and rate ratios for people of Chinese origin versus White people**

	**Chinese origin**			**White**			**Chinese versus White**	
**Sex/age group**	**Self-harm (n = 45)**	**Person- years**	**Rate/1000**	**Self-harm (n = 7111)**	**Person- years**	**Rate/ 1000**	**Rate ratio**	**(95% CI)**
Males and females combined								
16-34	37	58198	0.6	4408	1158943	3.8	0.17	(0.12, 0.23)
35-64	8	20496	0.4	2703	1046052	2.6	0.15	(0.08, 0.30)
16-64	45	78693	0.6	7111	2204995	3.2	0.18	(0.13, 0.24)
Males								
16-34	10	28969	0.3	1832	583614	3.1	0.11	(0.06, 0.20)
35-64	0	10218	0.0	1298	531299	2.4	0.00	-
16-64	10	39187	0.3	3130	1114913	2.8	0.09^a^	(0.05, 0.17)
Females								
16-34	27	29229	0.9	2576	575330	4.5	0.21	(0.14, 0.30)
35-64	8	10278	0.8	1405	514752	2.7	0.29	(0.14, 0.57)
16-64	35	39507	0.9	3981	1090082	3.7	0.24^a^	(0.17, 0.34)

^a^P value for sex interaction = 0.006.

### Characteristics

Analyses of socio-demographic characteristics, clinical characteristics, management, and repetition of self-harm were based on data for 96 people of Chinese origin and 19,856 White people with complete information on sex and age (Figure 1). People of Chinese origin who self-harmed were younger (mean age 28 years versus 33 years) and were more likely to be female (71% versus 55%) than White people (Table 2). Compared with White people they were also more likely to engage in self-injury (26% versus 17%) and self-poison using non-drugs (5.2% versus 0.7%) but were less likely to self-poison using drugs (69% versus 83%). The main method of self-injury used by people of Chinese origin was laceration (self-cutting) (88%). Compared to White people, people of Chinese origin were more likely to be married/partnered and less likely to be unemployed, and they were more likely to be students (44% versus 11%), with this difference more marked in males (41% versus 6%) than in females (45% versus 14%) (P for interaction = 0.004). People of Chinese origin who self-harmed were also more likely to report relationship problems with boyfriend/girlfriend or partner. Compared to White people, they were less likely to have clinical characteristics known to increase risk of suicidality, such as misuse of drugs/alcohol, previous or current psychiatric treatment, or previous self-harm, and they were less likely to report feeling depressed or experiencing suicidal thoughts or appetite problems. Logistic regression analysis showed very similar patterns of between-group differences in characteristics even after adjusting for sex and age (Additional file 1: Table S1); for example, people of Chinese origin who self-harmed had higher odds for relationship problems than White people (sex-age-adjusted odds ratio = 1.62, 95% CI 1.00-2.62), indicating that the difference could not be explained by a higher proportion of females or younger age in the Chinese group.

**Table 2:** **Socio-demographic characteristics, method of self-harm, precipitating factors, and clinical characteristics: people of Chinese origin versus White people**

	**Chinese origin**		**White**				
	**(N=96)**		**(N=19856)**				
**Characteristics** ^**a**^	**n**	**%**	**n**	**%**	**χ** ^**2**^	**df**	**p value** ^**b**^
Female	68	70.8	11007	55.4	7.8	1	**0.005**
Age					17.3	2	**<0.001**
15–24	49	51.0	7247	36.5			
25–34	30	31.3	5038	25.4			
35+	17	17.7	7571	38.1			
Self-harm method					19.9		**<0.001** ^**c**^
Self-poisoning (drugs)	66	68.8	16379	82.6			
Self-poisoning (others)	5	5.2	142	0.7			
Self-injury	25	26.0	3318	16.7			
Self-injury method					3.2	1	0.07
Laceration	14	87.5	1075	66.3			
Others	2	12.5	547	33.7			
Marital status					5.9	1	**0.01**
Not married or partnered	56	60.9	13792	72.3			
Married/partnered	36	39.1	5289	27.7			
Living status					2.0	2	0.37
With others	53	73.6	10011	66.2			
Alone	12	16.7	3515	23.2			
Homeless or hostel/lodgings or others	7	9.7	1600	10.6			
Employment status					109.8	3	**<0.001**
Employed	27	30.3	5612	29.8			
Student	39	43.8	1982	10.5			
Unemployed	11	12.4	6914	36.7			
Other	12	13.5	4323	23.0			
Precipitant factors							
Relationship problems with boy/girl-friend/partner	43	60.6	7563	47.8	4.6	1	**0.03**
Relationship problems with family	15	21.1	2805	17.7	0.6	1	0.46
Employment or work problems	10	14.1	2130	15.6	0.0	1	0.88
Financial problems	10	14.1	1795	11.4	0.5	1	0.47
Clinical characteristics							
Substance misuse	3	4.2	2412	15.0	6.5	1	**0.01**
Current alcohol misuse	7	9.6	5442	34.0	19.4	1	**<0.001**
Any previous psychiatric history	19	26.0	7388	45.2	10.7	1	**0.001**
Current psychiatric treatment	10	14.3	6239	37.8	16.4	1	**<0.001**
Any previous self-harm episode	31	40.8	8714	52.1	3.9	1	**0.05**
Symptoms of depression							
Feeling depressed	40	54.8	11125	67.2	5.1	1	**0.02**
Looks depressed	30	41.1	7507	45.7	0.6	1	0.43
Feeling hopeless	21	31.8	5937	36.9	0.7	1	0.40
Suicidal thoughts	17	23.9	5919	36.0	4.4	1	**0.03**
Suicidal plans	5	7.1	2187	13.4	2.4	1	0.12
Sleep problems	36	50.7	9455	57.8	1.5	1	0.23
Appetite problems	22	32.4	7204	44.3	3.9	1	**0.05**
Circumstances of episode							
Wanted to die	36	48.0	8745	56.4	2.1	1	0.14
Suicide note	9	12.2	1711	10.9	0.1	1	0.72
Avoiding discovery	11	14.9	1872	11.8	0.6	1	0.42
Premeditated	17	22.7	3341	20.7	0.2	1	0.67

^a^The numbers do not sum up to the total number due to missing data.

^b^p values < 0.05 are highlighted in bold.

^c^Fisher exact test.

### Risk assessment, clinical management, and outcome

There was no statistical evidence for a difference in receiving an assessment (from either emergency department or mental health professional) between people of Chinese origin and White people who presented with self-harm (Table 3), although there was a trend that people of Chinese origin were less likely to be assessed by mental health professional than White people (42% versus 53%, P = 0.053). People of Chinese origin who harmed themselves tended to be rated by clinicians as having lower risk for suicide and self-harm repetition than their White counterparts, and they were more likely to be referred to their GPs. There was no statistical evidence for a difference in risk of self-harm repetition within 12 months between the two groups (Chinese origin: 8%; White people: 14%; P = 0.13). The results were similar after adjusting for sex and age, although the difference in being assessed as with moderate/high suicide risk became statistically insignificant (Table 3).

**Table 3:** **Risk assessment, provision of services on discharge from the emergency department (ED), and repetition of self-harm: prevalence rate ratios (PRR) for people of Chinese origin versus White people**

	**Chinese origin**		**White**							
	**(N=96)**		**(N=19856)**		**Unadjusted**			**Sex-age-adjusted**		
**Variable**	**n**	**%**	**n**	**%**	**PPR** ^**a**^	**(95% CI)** ^**a**^	**p value** ^**a**^	**PPR** ^**a**^	**(95% CI)** ^**a**^	**p value** ^**a**^
Speciality of assessor										
ED clinician^b^	53	55.2	11595	59.8	0.92	(0.77, 1.11)	0.38	0.90	(0.75, 1.08)	0.27
Mental health professional^b^	40	41.7	10202	52.7	0.79	(0.62, 1.00)	0.05	0.80	(0.63, 1.02)	0.08
Either ED or mental health clinicians	78	81.3	17327	89.4	0.91	(0.83, 1.00)	0.05	0.91	(0.82, 1.00)	0.05
Risk Assessments:										
Medical (moderate/high)	35	46.7	8867	53.5	0.87	(0.68, 1.11)	0.27	0.91	(0.72, 1.17)	0.47
Suicide (moderate/high)	16	22.5	5751	34.8	**0.65**	**(0.42, 1.00)**	**0.05**	0.74	(0.49, 1.14)	0.17
Future self-harm (mod./high)	33	44.6	9718	58.4	**0.76**	**(0.59, 0.99)**	**0.04**	**0.77**	**(0.59, 1.00)**	**0.05**
ED outcome										
Self-discharge	0	0.0	549	3.8	-	-		-	-	
Discharged: no referral	7	8.8	1443	10.0	0.87	(0.43, 1.77)	0.71	0.73	(0.34, 1.57)	0.41
Referred: (i) To GP	14	27.5	1704	15.4	**1.78**	**(1.14, 2.78)**	**0.01**	**1.62**	**(1.04, 2.54)**	**0.03**
(ii) To Med/Surg service	18	22.5	4821	33.5	0.67	(0.45, 1.01)	0.06	0.73	(0.49, 1.10)	0.14
(iii) To mental health	38	47.5	5484	38.1	1.25	(0.99, 1.57)	0.06	1.25	(0.99, 1.57)	0.07
Self-harm repetition										
Within 12 months	7	8.0	2574	13.9	0.58	(0.28, 1.18)	0.13	0.62	(0.30, 1.26)	0.19

^a^Prevalence rate ratios that reach statistical significance (P < 0.05) are highlighted in bold.

^b^Not mutually exclusive. Some subjects were assessed by both emergency department clinicians and mental health professionals.

## Discussion

Our study showed that people of Chinese origin in the UK had less than one fifth the incidence of self-harm versus White people, and incidence was particularly low amongst men in this ethnic group. Compared with White people, people of Chinese origin who self-harmed were younger and more likely to be female and students and describe relationship problems as a precipitant to self-harm, and they were more likely to self-injure but less likely to self-poison with drugs compared to White people. They were less likely to have clinical risk factors such as drug/alcohol misuse and receiving psychiatric treatment, and were rated to have lower risk of self-harm repetition by treating clinicians. There was no statistical evidence for differences in likelihood of receiving assessment or self-harm repetition between people of Chinese origin and White people.

### Strengths and limitations

This is the first detailed analysis of self-harm amongst people of Chinese origin in the UK. The study was conducted in City of Manchester, which has one of the largest Chinese communities in the UK. There are several limitations to our study. First, the total number of people of Chinese origin who self-harmed was relatively small, which precluded more detailed analyses. Second, we only collected data on those who self-harmed and presented to hospital, and not in the community, and this may in part explain the comparatively low incidence of self-harm found in people of Chinese origin. One possible bias is that people of Chinese origin might be more likely to be classified as ‘ethnicity unknown’ than White people, although there is no obvious reason to assume this was the case in our study. Third, although we estimated that this cohort captured over 90% of self-harm presentations by residents living in City of Manchester [12], hospital attendances outside of the study areas may introduce bias to our estimates. Fourth, Chinese people living in the UK are heterogeneous, born in the UK or arriving at different ages, or coming from various countries with differing social values and experiences in psychological adjustment to the migratory process [17]. Thus, the rate and pattern of self-harm may differ by subgroup. Finally, people of Chinese origin living in Manchester may not be representative of the overall Chinese population living in England, as in recent years the Chinese population living in City of Manchester was overrepresented with international students from China. The University of Manchester is amongst the largest recruiters of international students amongst UK universities [18], and in 2013–2014 there are around 4500 international students from China at the University of Manchester (statistics provided by the University of Manchester).

### Comparison with existing literature

There is limited published evidence concerning self-harm episodes presenting to hospitals in China because population-level self-harm surveillance systems do not exist, as is the situation in most countries. A retrospective analysis of emergency department admissions in Hong Kong in 1997–2003 showed low incidence of self-harm (0.3 per 1000 person-years) that was similar to our data [19]. In contrast, a study based on a prospective surveillance system of emergency department admissions resulted from self-harm in a large Taiwanese city showed a much higher incidence of self-harm (~1.5 per 1000 person-years) in 2004–2006 [20].

Our data showed lower self-harm incidence amongst people of Chinese origin than in White people in England. This may truly reflect a lower frequency of self-harm, or alternatively it may reflect markedly different post-episode help-seeking behaviours in this ethnic group. A study from Birmingham, England, shows that most Chinese people prefer to seek help from their family or friends when having issues around mental health [17]. Previous studies also show that health service use amongst Chinese people living in the UK is amongst the lowest of all social groups [21]. People of Chinese origin may be less likely than White people to present to hospital following a self-harm episode, with the exception perhaps of the most severe cases, due to issues associated with stigma around mental illness and the perception that services would not be appropriate for their needs [22]. However, our data did not appear to support this notion, as people of Chinese origin were assessed as presenting with lower medical seriousness compared to White people, and with low risk of repetition or suicide. Other practical barriers, such as time, knowledge of access, and language, may be more important factors for lower use of mental health service use in the Chinese group [23]. Before self-harm incidence can be assumed to be lower amongst the Chinese group in the UK, further research is needed to better understand the attitudes towards self-harm and the role of statutory services within their community.

If the lower self-harm incidence we observed in people of Chinese origin truly reflects a low frequency of occurrence, this may be explained by traditional Chinese values that discourage self-harm, a healthy migrant effect, choice of method and access to means, or a strong local social network. Confucius, one of the most influential Chinese philosophers, is quoted as saying “shen ti fa fu, shou zhi fu mu, bu gan hui shang” (“My whole body, including my hair and skin, is given by my parents. I dare not harm it”) [24]. These traditional values may reduce the risk of self-harm amongst people of Chinese origin if they hold a strong belief in them. A study of adolescents in Hong Kong showed that girls, but not boys, who placed a higher value on obedience and respect for elders were less likely to report suicidal ideation or suicide attempt in the last year [25]. By contrast, the ‘healthy migrant hypothesis’ argues that migration is a selection process that filters away the vulnerable and keeps the healthy [26], and these ‘healthy migrants’ may thus present reduced risk of poor mental health and self-harm [27]. As mentioned above, in recent years a significant proportion of people of Chinese origin living in City of Manchester were international students from China; these students would be mostly healthy and are more likely to come from an advantaged socioeconomic background and thus have lower risk of self-harm.

Self-poisoning is more likely than self-injury (self-cutting in particular) to result in hospital presentations for treatment, whereas people of Chinese origin in this study were more likely to self-injure than White people. Studies in China, consistent with our findings, show that self-poisoning using drugs (usually anxiolytics or sleeping pills) was the most common method of self-harm [28], but people of Chinese origin in the UK may have reduced accessibility to these drugs compared to White people as they are less likely to seek help from GPs [22]. One distinct feature of self-harm in China is that a considerable proportion of people who self-harmed used pesticide ingestion (e.g. 28% in Phillip and Yang [28]), and ready access to toxic pesticide led to elevated suicide risk, particularly in rural areas [3,29]. In contrast our data indicated that few people of Chinese origin deliberately ingested non-drug substances, which could be explained by limited access to pesticides in the urban study population. Lastly, the proportion of residents who are of Chinese origin is relatively high in Manchester compared to other areas in the UK, and this may lead to a stronger social network in the Chinese group locally. It has been shown that self-harm incidence among minority groups is lower in areas where there is a relatively high ethnic minority density [30].

Compared to findings from several studies based on hospital presentations for self-harm in China, which showed a mean age around 31–33 years [31-33], people of Chinese origin in our study appeared to be somewhat younger (mean age 28 years) and overrepresented by students (44%). By contrast only 12% of suicide attempters were students in one study from an urban area in China [32]. Our finding reflects the composition of the Chinese population living in Manchester – as mentioned above people of Chinese origin living in City of Manchester were overrepresented with international students from China in recent years.

The clinical characteristics of people of Chinese origin who self-harmed in our study were generally similar to findings from China. Suicide attempts in China were often impulsive, characterised by a short time period between planning and attempt [28]; they were commonly in response to interpersonal conflict [31,34,35], and were less likely to be associated with psychiatric disorders compared to findings from most Western countries [32]. Compared to White people, people of Chinese origin who self-harmed in our study were less likely to present clinical risk factors or receive psychiatric treatment, and thus were rated to have lower risk of suicide or self-harm repetition. This suggests a higher level of impulsivity for self-harm in the Chinese group compared to White people, although we did not directly measure impulsivity. However, people of Chinese origin who self-harmed in our study did not differ significantly from White people in the likelihood of leaving suicide notes, avoiding discovery, or being premeditated, which were all indicators of planned suicide or low impulsivity, and therefore whether self-harming behaviours in people of Chinese origin are more impulsive needs further research. Some characteristics of the Chinese group, such as low level of psychopathology and an association with relationship problems, were similarly seen in the South Asian ethnicity group [5,15]. Although the number of people of Chinese origin who repeated self-harm was small in our study (n = 7), the 12 month repetition rate (8%) was similar to that reported in Taiwan (6-9%) [36,37]. In line with findings from Taiwan, our data suggested that self-harm repetition rate in people of Chinese origin was lower than that found in White people (14%), although the difference was not statistically significant (p = 0.13).

### Implications

Future research needs to better understand the low incidence of self-harm presenting to hospitals amongst people of Chinese origin, and investigate whether this masks a large number of hidden episodes in the community due to less help-seeking or barriers to services [22]. The likelihood and reason for undisclosed self-harm and less frequent help seeking could be studied using qualitative methodology such as focus groups or in-depth interviews with people who self-harmed. Alternatively this ethnic population may have protective factors that could inform public health initiatives. Future research also needs to investigate risk and protective factors in different subgroups of people of Chinese origin [17], e.g. students. It has been also shown that suicide risk may differ in first- and second-generation Chinese immigrants, as well as between first-generation immigrants who arrived at different ages [38].

Our findings have implications for the assessment and treatment of self-harm amongst people of Chinese origin in the UK. Relevant healthcare professionals need to be aware of the risk characteristics and be sensitive to the specific need in this ethnic group. GPs have been shown to play a pivotal role in the management of mental health problems amongst people of Chinese origin living in the UK [22]. Student counselling services providing interventions for interpersonal problems should be accessible to students within this ethnic group and be readily referred by treating clinicians. Psychological interventions of improving problem solving and interpersonal skills could be particularly helpful [34].

## Conclusions

People of Chinese origin in the UK appear to have much lower incidence of self-harm than White people, particularly in men. There are some distinct characteristics of self-harm in the Chinese group, such as a younger age, a higher proportion of females and students, and increased likelihood of reporting relationship problems as a precipitant to self-harm and using self-injury as the method of self-harm. They are also less likely to present clinical risk factors such as drug/alcohol misuse and receiving psychiatric treatment. Healthcare professions need to be aware of these characteristics and attend to specific needs amongst people who have self-harmed in this ethnic group.

## Additional file

Additional file 1: Table S1. Logistic regression analysis of socio-demographic characteristics, method of self-harm, precipitating factors, and clinical characteristics amongst people of Chinese origin (N = 96) vs. White people (N = 19856, comparison group).

## Abbreviations

MaSH project: Manchester Self-Harm project
CI: Confidence Interval

## References

[1] ESRC Centre on Dynamics of Ethnicity (CoDE): How has ethnic diversity grown 1991-2001-2011? 2012, https://www.google.com.tw/url?sa=t&rct=j&q=&esrc=s&source=web&cd=1&cad=rja&uact=8&ved=0CB4QFjAA&url=http%3A%2F%2Fwww.ethnicity.ac.uk%2Fmedialibrary%2Fbriefings%2Fdynamicsofdiversity%2Fhow-has-ethnic-diversity-grown-1991-2001-2011.pdf&ei=Y5QtVdPJD8788AWgmIHIAg&usg=AFQjCNFJtDqKAF-f9x_mcIjo06GOHKU3lQ&sig2=pM1-MHC6cQnRRu_f1iE8jw. Retrieved on 9 May 2014.

[2] Universities UK. The UK’s relationship with China: Universities. 2013, http://www.universitiesuk.ac.uk/highereducation/Documents/2013/UKandChina.pdf. Retrieved on 19 July 2014.

[3] Phillips MR, Li X, Zhang Y (2002). Suicide rates in China, 1995–99. Lancet.

[4] Bhugra D, Gupta S, Bhui K, Craig T, Dogra N, Ingleby JD (2011). WPA guidance on mental health and mental health care in migrants. World Psychiatry.

[5] Bhui K, McKenzie K, Rasul F (2007). Rates, risk factors & methods of self harm among minority ethnic groups in the UK: a systematic review. BMC Public Health.

[6] Shah A, Lindesay J, Dennis M (2011). Suicides by country of birth groupings in England and Wales: age-associated trends and standardised mortality ratios. Soc Psychiatry Psychiatr Epidemiol.

[7] Cooper J, Steeg S, Webb R, Stewart SL, Applegate E, Hawton K (2013). Risk factors associated with repetition of self-harm in black and minority ethnic (BME) groups: A multi-centre cohort study. J Affect Disord.

[8] Bickley H, Steeg S, Turnbull P, Haigh M, Donaldson I, Matthews V, et al. Self-Harm in Manchester January 2010 to December 2011. 2013, http://www.bbmh.manchester.ac.uk/cmhr/research/centreforsuicideprevention/MaSH/reports/MASHREPORT1011.pdf. Retrieved on 19 July 2014.

[9] Cooper J, Kapur N, Webb R, Lawlor M, Guthrie E, Mackway-Jones K (2005). Suicide after deliberate self-harm: a 4-year cohort study. Am J Psychiatry.

[10] Hawton K, Harriss L, Hall S, Simikin S, Bale E, Bond A (2003). Deliberate self-harm in Oxford, 1990–2000: a time of change in patient characteristics. Psychol Med.

[11] Bergen H, Hawton K, Waters K, Cooper J, Kapur N (2010). Epidemiology and trends in non-fatal self-harm in three centres in England: 2000–2007. Br J Psychiatry.

[12] Kapur N, Steeg S, Webb R, Haigh M, Bergen H, Hawton K (2013). Does clinical management improve outcomes following self-harm? Results from the multicentre study of self-harm in England. PLoS One.

[13] Census 2011. Ethnic group by sex by age. 2011. http://www.nomisweb.co.uk/census/2011/dc2101ew. Retrieved on 19 July 2014.

[14] Office for National Statistics. Published ad hoc data and analysis: Population, requests during June 2012. 2012, http://www.ons.gov.uk/ons/about-ons/business-transparency/freedom-of-information/what-can-i-request/published-ad-hoc-data/pop/june-2012/index.html. Retrieved on 19 July 2014.

[15] Cooper J, Husain N, Webb R, Waheed W, Kapur N, Guthrie E (2006). Self-harm in the UK: differences between South Asians and Whites in rates, characteristics, provision of service and repetition. Soc Psychiatry Psychiatr Epidemiol.

[16] Cooper J, Murphy E, Webb R, Hawton K, Bergen H, Waters K (2010). Ethnic differences in self-harm, rates, characteristics and service provision: three-city cohort study. Br J Psychiatry.

[17] Huang SL, Spurgeon A (2006). The mental health of Chinese immigrants in Birmingham, UK. Ethn Health.

[18] UK Council for International Student Affairs. Top 20 largest recruiters of international students 2012–13. 2014, http://www.ukcisa.org.uk/Info-for-universities-colleges--schools/Policy-research--statistics/Research--statistics/International-students-in-UK-HE/#Top-20-largest-recruiters-of-international-students-2012-13. Retrieved on 19 July 2014.

[19] The Hong Kong Jockey Club Centre for Suicide Research and Prevention, The University of Hong Kong. Attempted Suicide Rates (A&E Admission) by Gender. 1997–2003, http://csrp.hku.hk/WEB/eng/statistics.asp?id=208#1. Retrieved on 20 May 2014.

[20] Kuo CJ, Gunnell D, Chen CC, Yip PS, Chen YY (2012). Suicide and non-suicide mortality after self-harm in Taipei City, Taiwan. Br J Psychiatry.

[21] Sproston K, Pitson L, Whitfield G, Walker E (1999). Health and lifestyles of the Chinese population in England.

[22] Li PL, Logan S, Yee L, Ng S (1999). Barriers to meeting the mental health needs of the Chinese community. J Public Health Med.

[23] Kung WW (2004). Cultural and practical barriers to seeking mental health treatment for Chinese Americans. J Community Psychol.

[24] Liu W (2013). Moralized hygiene and nationalized body: anti-cigarette campaigns in china on the eve of the 1911 revolution. Cross-Currents: East Asian History and Culture Rev.

[25] Lam TH, Stewart SM, Yip PSF, Leung GM, Ho LM, Ho SY (2004). Suicidality and cultural values among Hong Kong adolescents. Soc Sci Med.

[26] Marmot MG, Adelstein AM, Bulusu L (1984). Lessons from the study of immigrant mortality. Lancet.

[27] Kwan YK, Ip WC (2007). Suicidality and migration among adolescents in Hong Kong. Death Stud.

[28] Phillips MR, Yang GH (2004). Suicide and attempted suicide–China, 1990–2002. MMWR Morb Mortal Wkly Rep.

[29] Eddleston M, Gunnell D (2006). Why suicide rates are high in China. Science.

[30] Neeleman J, Wilson-Jones C, Wessely S (2001). Ethnic density and deliberate self harm; a small area study in south east London. J Epidemiol Community Health.

[31] Zhang J, Jia S, Jiang C, Sun J (2006). Characteristics of Chinese suicide attempters: an emergency room study. Death Stud.

[32] Bi B, Tong J, Liu L, Wei S, Li H, Hou J (2010). Comparison of patients with and without mental disorders treated for suicide attempts in the emergency departments of four general hospitals in Shenyang, China. Gen Hosp Psychiatry.

[33] Zhao P, Yang R, Phillips MR (2010). Age-specific characteristics of serious suicide attempters in China. Suicide Life Threat Behav.

[34] Li X, Phillips MR, Cohen A (2012). Indepth interviews with 244 female suicide attempters and their associates in northern China: understanding the process and causes of the attempt. Crisis.

[35] Wei S, Liu L, Bi B, Li H, Hou J, Chen W (2013). Comparison of impulsive and nonimpulsive suicide attempt patients treated in the emergency departments of four general hospitals in Shenyang, China. Gen Hosp Psychiatry.

[36] Chen VC, Tan HK, Cheng AT, Chen CY, Liao LR, Stewart R (2010). Non-fatal repetition of self-harm: population-based prospective cohort study in Taiwan. Br J Psychiatry.

[37] Kwok CL, Yip PS, Gunnell D, Kuo CJ, Chen YY (2014). Non-fatal repetition of self-harm in Taipei City, Taiwan: cohort study. Br J Psychiatry.

[38] Zhang J, Fang L, Wu YW, Wieczorek WF (2013). Depression, anxiety, and suicidal ideation among Chinese Americans: a study of immigration-related factors. J Nerv Ment Dis.

