# Supplementary material for: Self-harm amongst people of Chinese origin versus White people living in England: a cohort study
Source: BMC Psychiatry. 2015 Apr 14;15:79. doi: 10.1186/s12888-015-0467-0 (PMC4409751; doi:10.1186/s12888-015-0467-0)
Supplement: Additional file 1: Table S1. — Logistic regression analysis of socio-demographic characteristics, method of self-harm, precipitating factors, and clinical characteristics amongst people of Chinese origin (N = 96) vs. White people (N = 19856, comparison group). [file 12888_2015_467_MOESM1_ESM.doc]

**Additional file 1: Table S1.** Logistic regression analysis of socio-demographic characteristics, method of self-harm, precipitating factors, and clinical characteristics amongst people of Chinese origin (N = 96) vs. White people (N = 19856, comparison group)

|  | **Chinese vs White** | | | | **Chinese vs White** | | | |
| --- | --- | --- | --- | --- | --- | --- | --- | --- |
|  | **Unadjusted** | | | | **Sex-age-adjusted** | | | |
| **Characteristics^a^** | **OR^a^** | **(95% CI)** | | **p** | **OR^a^** | **(95% CI)** | | **p** |
| Female | **1.95** | **(1.26** | **, 3.03)** | **0.003** | **1.86** | **(1.19** | **, 2.89)** | **0.006** |
| Age |  |  |  |  |  |  |  |  |
| 15–24 | 1.00 |  |  |  | 1.00 |  |  |  |
| 25–34 | 0.88 | (0.56 | , 1.39) | 0.58 | 0.95 | (0.60 | , 1.50) | 0.82 |
| 35+ | **0.33** | **(0.19** | **, 0.58)** | **<0.001** | **0.35** | **(0.20** | **, 0.62)** | **<0.001** |
| Self-harm method |  |  |  |  |  |  |  |  |
| Self-poisoning (drugs) | 1.00 |  |  |  | 1.00 |  |  |  |
| Self-poisoning (other) | **8.74** | **(3.47** | **, 22.01)** | **<0.001** | **11.45** | **(4.49** | **, 29.21)** | **<0.001** |
| Self-injury | **1.87** | **(1.18** | **, 2.97)** | **0.008** | **1.91** | **(1.20** | **, 3.04)** | **0.007** |
| Self-injury method |  |  |  |  |  |  |  |  |
| Laceration | 1.00 |  |  |  | 1.00 |  |  |  |
| Others | 3.56 | (0.81 | , 15.73) | 0.094 | 3.28 | (0.74 | , 14.66) | 0.12 |
| Marital status |  |  |  |  |  |  |  |  |
| Not married or partnered | 1.00 |  |  |  | 1.00 |  |  |  |
| Married/partnered | **1.68** | **(1.10** | **, 2.55)** | **0.016** | **1.96** | **(1.27** | **, 3.03)** | **0.002** |
| Living status |  |  |  |  |  |  |  |  |
| With others | 1.00 |  |  |  | 1.00 |  |  |  |
| Alone | 0.64 | (0.34 | , 1.21) | 0.17 | 0.84 | (0.44 | , 1.61) | 0.61 |
| Homeless or hostel / lodgings or others | 0.83 | (0.38 | , 1.82) | 0.64 | 0.84 | (0.38 | , 1.84) | 0.66 |
| Employment status |  |  |  |  |  |  |  |  |
| Employed | 1.00 |  |  |  | 1.00 |  |  |  |
| Student | **4.09** | **(2.50** | **, 6.70)** | **<0.001** | **5.21** | **(2.86** | **, 9.47)** | **<0.001** |
| Unemployed | **0.33** | **(0.16** | **, 0.67)** | **0.002** | **0.34** | **(0.17** | **, 0.68)** | **0.002** |
| Other | 0.58 | (0.29 | , 1.14) | 0.11 | 0.60 | (0.30 | , 1.19) | 0.15 |
| Precipitant relationship factors |  |  |  |  |  |  |  |  |
| Relationship problems with boy/girl-friend/partner | **1.67** | **(1.04** | **, 2.70)** | **0.034** | **1.62** | **(1.00** | **, 2.62)** | **0.048** |
| Relationship problems with family | 1.24 | (0.70 | , 2.20) | 0.46 | 1.00 | (0.56 | , 1.79) | 1.00 |
| Employment or work problems | 1.05 | (0.54 | , 2.06) | 0.88 | 1.01 | (0.51 | , 1.97) | 0.99 |
| Financial problems | 1.28 | (0.65 | , 2.50) | 0.47 | 1.48 | (0.75 | , 2.90) | 0.26 |
| Clinical characteristics |  |  |  |  |  |  |  |  |
| Substance misuse | **0.25** | **(0.08** | **, 0.79)** | **0.019** | **0.23** | **(0.07** | **, 0.74)** | **0.013** |
| Current alcohol misuse | **0.21** | **(0.09** | **, 0.45)** | **<0.001** | **0.24** | **(0.11** | **, 0.53)** | **<0.001** |
| Any previous psychiatric history | **0.43** | **(0.25** | **, 0.72)** | **0.001** | **0.47** | **(0.28** | **, 0.80)** | **0.006** |
| Current psychiatric treatment | **0.27** | **(0.14** | **, 0.54)** | **<0.001** | **0.30** | **(0.15** | **, 0.59)** | **<0.001** |
| Any previous self-harm episode | 0.63 | (0.40 | , 1.00) | 0.050 | 0.64 | (0.40 | , 1.01) | 0.055 |
| Symptoms of depression |  |  |  |  |  |  |  |  |
| Feeling depressed | **0.59** | **(0.37** | **, 0.94)** | **0.026** | **0.63** | **(0.39** | **, 1.00)** | **0.049** |
| Looks depressed | 0.83 | (0.52 | , 1.32) | 0.44 | 0.89 | (0.56 | , 1.43) | 0.64 |
| Feeling hopeless | 0.80 | (0.48 | , 1.34) | 0.40 | 0.87 | (0.51 | , 1.46) | 0.59 |
| Suicidal thoughts | **0.56** | **(0.32** | **, 0.97)** | **0.038** | 0.60 | (0.35 | , 1.04) | 0.066 |
| Suicidal plans | 0.50 | (0.20 | , 1.24) | 0.13 | 0.56 | (0.22 | , 1.40) | 0.21 |
| Sleep problems | 0.75 | (0.47 | , 1.20) | 0.23 | 0.82 | (0.51 | , 1.31) | 0.40 |
| Appetite problems | 0.60 | (0.36 | , 1.00) | 0.051 | 0.65 | (0.39 | , 1.08) | 0.097 |
| Circumstances of episode |  |  |  |  |  |  |  |  |
| Wanted to die | 0.71 | (0.45 | , 1.12) | 0.15 | 0.79 | (0.50 | , 1.24) | 0.31 |
| Suicide note | 1.13 | (0.56 | , 2.28) | 0.73 | 1.22 | (0.60 | , 2.45) | 0.58 |
| Avoiding discovery | 1.30 | (0.68 | , 2.47) | 0.42 | 1.44 | (0.76 | , 2.74) | 0.27 |
| Premeditated | 1.13 | (0.65 | , 1.94) | 0.67 | 1.20 | (0.70 | , 2.06) | 0.51 |

^a^ Odds ratios that reach statistical significance (P < 0.05) are highlighted in bold.
